# A Single‐Center, Open Label, Study to Evaluate the Effectiveness of Acne Scar Gel (ASG) in Improving Acne Scar Appearance and Reducing Hyperpigmentation in Subjects With Mild‐To‐Moderate Acne Scars

**DOI:** 10.1111/jocd.70081

**Published:** 2025-03-07

**Authors:** Bruce E. Katz, Mithun Mishu, Madeline Malayeva

**Affiliations:** ^1^ JUVA Skin & Laser Center New York New York USA

**Keywords:** acne, acne scarring, acne vulgaris, hyperpigmentation, postinflammatory hyperpigmentation, topical acne therapy

## Abstract

**Background:**

Acne vulgaris affects up to 80% of individuals aged 11 to 30 years and is one of the most common diseases in the world. Acne and its sequelae, such as redness, hyperpigmentation, irritation, and scarring, can have a deleterious effect on quality of life. Topical therapies are the standard of care, and compliance is central to success.

**Aims:**

The purpose of the study was to evaluate the efficacy of a novel topical formulation (TASG) for the improvement of acne scar appearance and for reducing hyperpigmentation.

**Patients/Methods:**

This single‐center, open‐label clinical trial included healthy subjects (*n* = 27, age range 18 to 45 years) presenting with mild to moderate acne scarring and hyperpigmentation. Study duration was 4 visits over a 12‐week period. Product was to be applied twice daily.

**Results:**

No significant treatment‐related adverse events were noted. Data show that 72.5% of subjects had smoother skin and reduced hyperpigmentation (as per observer grading of photographs) and redness at the final week 12 follow‐up visit; subjective questionnaire results showed high patient satisfaction, with overall positive impressions of the product itself and its regular use.

**Conclusions:**

The data show that TASG is a safe and effective therapeutic option for improving the appearance of acne scars and related hyperpigmentation.

## 1 Introduction

Acne vulgaris is a ubiquitous condition affecting up to 80% of individuals aged 11 to 30 years [1] and is one of the most common diseases in the world [2]. The chronic obstructive and inflammatory disease is caused by over secretion by the sebaceous glands. Acne and its sequelae can have a dramatic effect on quality of life, including contributing to higher social appearance anxiety [3].

The condition brings with it a significant likelihood of scarring, thought to be the result of a suboptimal wound healing response due to acne‐associated inflammation [4]. Current therapies for acne scarring are numerous and include ablative and nonablative lasers, microneedling with and without adjunctive topicals such as platelet‐rich plasma, radiofrequency, excision, and trichloroacetic acid (TCA CROSS) [4].

Acne Scar Gel (ASG) with ReTex‐5 technology (patent pending) from Newmedical Technology Inc. (Northbrook, IL., USA) is formulated as a noninvasive topical therapy for acne scarring. The purpose of the study was to evaluate the efficacy of the product for the improvement of acne scar appearance and the reduction of hyperpigmentation.

## 2 Study Methods

The efficacy of ASG was examined in a single center, open label clinical trial involving otherwise healthy subjects (*n* = 27, age range 18 to 45 years) presenting with mild to moderate acne scarring and dormant acne. A minimum of 60% of the subject group was to present with hyperpigmentation. The study was conducted under good clinical practice guidelines, and informed consent was obtained from all subjects as per the Declaration of Helsinki.

Exclusion criteria included (but was not limited to) the presence of any systemic disorder or facial dermatoses other than acne that would in any way confound the interpretation of the study results (e.g., atopic dermatitis, eczema, or psoriasis); a history of skin cancer; female subjects who are pregnant, expect to become pregnant, or are lactating; subjects who have started, ceased, or changed hormonal therapy of any kind within 3 months prior to the study; actual or planned excessive exposure to natural or artificial UV light (such as tanning, phototherapy, or a tropical vacation) within 1 month prior to the study; use of systemic drugs for more than three consecutive days related to antibiotics, anti‐inflammatory, or corticoids 4 weeks prior, and/or use of topical drugs for more than three consecutive days related to antibiotics, anti‐inflammatory, or corticoids in the 2 weeks prior to the study; or the presence of any condition or use of medication and/or a history of medical/surgical events which, in the opinion of the investigator, could compromise the safety of the subject or affect the outcome of the study. Additionally, the use of the product is contraindicated in patients with a known sensitivity to silicone, vitamin C, bakuchiol, or azelaic acid.

Study duration was four visits over a 12‐week period at baseline (week 0) and follow‐up at weeks 4, 8, and final follow‐up at week 12. At the baseline visit, subjects were given an adequate supply of the ASG product (as well as Silagen SPF +30 Gel in case of sun exposure) and instructed on the proper use of the products, as well as given a reference sheet to take home regarding product use during the study. Product was to be applied twice daily, once before bedtime and again in the morning, to all areas with acne scarring. When applying the morning dose of ASG product, if the subject planned to be exposed to the sun, they were instructed to wait 10 min and then apply Silagen + SPF 30 Gel.

The primary evaluation was 3D imaging photography (using Quantificare 3D LifeViz camera, Quantificare Inc., Suwanee, GA, USA). Results were measured by the percent of before and after photos correctly identified by the observers. A secondary patient questionnaire was also administered (subjective rating 1–5 where 1 = disagree, 2 = slightly disagree, 3 = neither agree nor disagree, 4 = slightly agree, 5 = agree) with 10 questions about hyperpigmentation and skin appearance (scores added for a total between 10 and 50) and six questions focused on impressions of the product and its use (score totals not relevant). These evaluations were performed at weeks 4, 8, and 12 (final follow‐up).

## 3 Results

Of 27 subjects, 23 completed the study and 4 were lost to follow‐up. Medical observer data show that 72.5% of subjects had smoother skin and reduced hyperpigmentation and redness at the final week 12 follow‐up visit; subjective participant assessment results showed high patient satisfaction and overall positive impressions of the product itself and its regular use. No significant treatment‐related adverse events were noted.

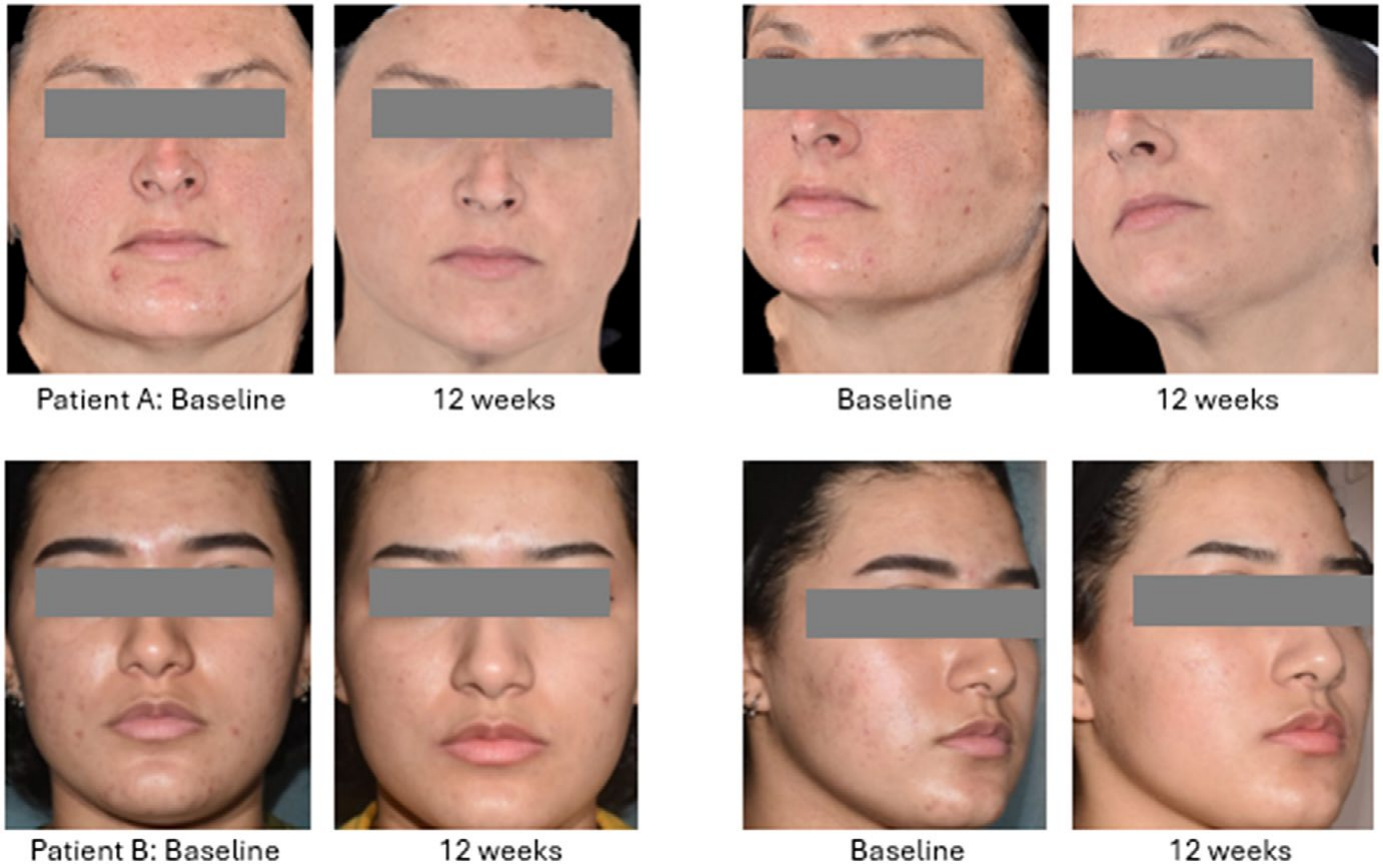

Patient perception of skin texture and coloration throughout the study duration was also rated via subjective questionnaire, and the results were averaged at each time point; total scores were also calculated for the 10 questions relating to skin improvement and the 6 questions relating to product use. Improvement was indicated by increases in average score from one time point rating to another later time point.

Analysis of the positive responses from the participants' self‐assessments shows a significant improvement in hyperpigmentation and overall skin appearance over the study period. Participants noticed a reduction in the color of hyperpigmentation spots and the appearance of their indented scars, making them feel more confident about their skin complexion. Additionally, they experienced overall enhancements in skin appearance, including improvements in texture, color evenness, and smoothness.

**FIGURE 1:**
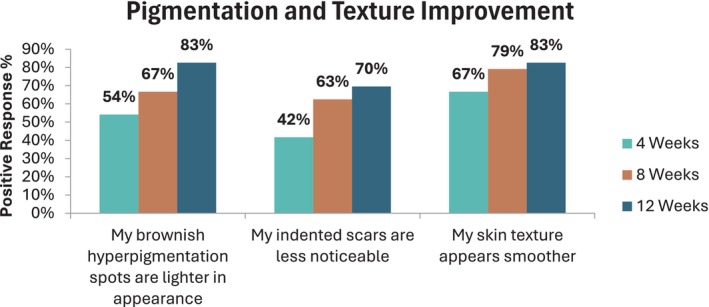
Skin pigmentation and texture improvement.

Up to 83% of the participants reported positive responses and observed a reduction in the appearance of hyperpigmentation spots and texture improvement between Day 0 and the end of the study period, with a majority indicating that the product provided a more even skin tone. Furthermore, the subjects reported a 70% improvement in the appearance of their indented scars and felt more confident about the appearance of their skin, as shown in Figure 1.

**FIGURE 2:**
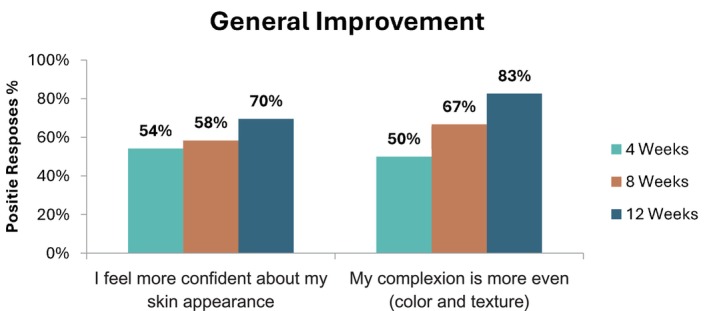
Skin general improvement.

Additionally, the data from the study participants showed an 83% improvement in skin complexion, including texture, color evenness, and smoothness after 12 weeks of treatment, as depicted in Figure 2.

Questions related to user experience at weeks 4, 8, and 12 reveal high patient satisfaction with the feel, application, and wear of the product. The scores were averaged and are listed in Table 1.

**TABLE 1:** Patient perception of product use.

Question	Average score
The product is easy to spread	4.16
Does the product dispense easily?	4.59
The product absorbs quickly	3.72
The scent of the product is pleasant	3.73
The product doesn't make my skin feel sticky	3.64
The product doesn't make my skin feel greasy	3.47

*Note:* Data captured at weeks 4, 8, and final follow‐up (week 12). Patients rated these questions on a subjective 1–5 where 1 = disagree, 2 = slightly disagree, 3 = neither agree nor disagree, 4 = slightly agree, 5 = agree.

A majority of participants expressed satisfaction with the treatment, citing reduced hyperpigmentation, enhanced indented acne scar appearance, and a smoother skin appearance as key outcomes.

## 4 Discussion

ASG with Retex‐5 Technology is a stable silicone gel‐based topical that is easy to apply and is absorbed quickly by the skin, formulated as a noninvasive therapy to improve the appearance of acne scarring and associated dark or red discoloration. Key active ingredients include 10% azelaic acid, acetyl zingerone, bakuchiol, vitamin C, licorice root extract, and medical‐grade silicone gel, all of which have been shown to be effective in treating symptoms of acne scars and/or hyperpigmentation. According to a 2002 consensus paper (International Clinical Recommendations on Scar Management) [5] silicone has been a top scar therapy since the 1980s. ReTex‐5 Technology allows the ingredients to remain active and stable within the product formulation. The product is meant to be used twice daily, in the morning and evening.

The study showed a high level of satisfaction (83%) regarding hyperpigmentation improvement, with a notable decline in neutral responses over time. This suggests that as participants continued to use the product over the 12‐week period, they became more convinced of its effectiveness. The self‐assessment data on the improvement of indented acne scars appearance, with a 70% agreement by the end of the study, suggests that the majority of participants noticed positive changes over time.

Similar to the data on hyperpigmentation, the number of undecided participants (neutral responses) declined as the study progressed. This reinforces the idea that as participants continued to use the product, their perception of improvement became clearer, as shown in Table 2. The expected variability in response times among individuals—some seeing faster results than others (hyper responders)—also aligns with the idea that gradual improvements become clearer as time goes on, as shown in Tables 3 and 4.

**TABLE 2:** Number of patients showing improvement in acne scars based on visual observation.

Medical Observer 1	16 out of 23 (69.6%)
Medical Observer 2	21 out of 23 (91.3%)
Medical Observer 3	13 out of 23 (56.5%)

**TABLE 3:** Pigmentation and texture improvement.

Weeks	Number of patient	Response	My brownish hyperpigmentation spots are lighter in appearance		My indented scars are less noticeable		My skin texture appears smoother	
4	24	3	6	25%	8	33%	5	21%
		4	8	54%	7	42%	10	67%
		5	5		3		6	
8	24	3	6	25%	5	21%	2	8%
		4	8	67%	8	63%	11	79%
		5	8		7		8	
12	23	3	3	13%	2	8%	4	17%
		4	10	83%	9	70%	9	83%
		5	9		7		10	
Neutral—Positive Responses at the end of the study			22	**96%**	18	**78%**	23	**100%**

*Note:* Bold values are statistically significant.

**TABLE 4:** General improvement.

Weeks	Number of patient	Response	My complexion is more even (color and texture)		I feel more confident about my skin appearance	
4	24	3	9	37.5%	8	33%
		4	8	50%	8	54%
		5	4		5	
8	24	3	6	25%	7	29%
		4	9	67%	7	58%
		5	7		7	
12	23	3	4	17%	6	26%
		4	13	83%	8	70%
		5	6		8	
Neutral—Positive Responses at the end of the study			23	**100%**	22	**96%**

*Note:* Bold values are statistically significant.

The findings reflected in Tables 5 and 6 indicate a strong trend toward improvement, as evidenced by the significant decrease in neutral and negative responses. This trend reinforces the anticipated outcome that extended use of the product enhances skin appearance, specifically pigmentation and texture. The scarcity of negative responses toward the end of the study further underscores the product's effectiveness over time.

**TABLE 5:** Decrease in percentage of the undecided (neither agree nor disagree) patient.

Weeks	Pigmentation and texture improvement			General improvement	
	My brownish hyperpigmentation spots are lighter in appearance	My indented scars are less noticeable	My skin texture appears smoother	My complexion is more even (color and texture)	I feel more confident about my skin appearance
4	25%	33%	21%	37.5%	33%
8	20%	21%	8%	25%	29%
12	13%	8%	17%	17%	26%

**TABLE 6:** Decrease in percentage of the negative responses patient (disagree or slightly disagree).

Weeks	Pigmentation and texture improvement			General improvement	
	My brownish hyperpigmentation spots are lighter in appearance	My indented scars are less noticeable	My skin texture appears smoother	My complexion is more even (color and texture)	I feel more confident about my skin appearance
4	21%	25%	12%	12.5%	13%
8	8%	16%	13%	8%	13%
12	4%	22%	0%	0%	4%

The product use questionnaire elicited positive responses across all questions. The average score consistently equaled or exceeded 3 (neutral), indicating an overall acceptance of product qualities that suggests a high likelihood of compliance, due to the lack of qualities that may “turn off” patients to regular product use. Given that the outcomes for improvement in skin coloration and texture become most readily apparent to patients after week 8 according to the data, this is essential because users must employ the product twice daily for a substantial length of time before results manifest. The highest rated answers were to the questions, “The product is easy to spread” (average 4.16) and “Does the product dispense easily?” (average 4.59) indicating the basic physical characteristics of the product during application are its strongest attributes regarding daily use. The lowest rated average was for the question, “The product doesn't make my skin feel greasy” (3.47), but this was still 0.47 points above neutral (score of 3 out of 5).

The results show that the product is safe for twice‐daily home use and effective for improving the appearance of acne scars, as well as reducing associated hyperpigmentation and redness. The product is convenient and easy to use. Thus, the ASG presents an attractive option to patients enduring acne scars and can expect visible improvement from regular, correct use of the product.

Although it was not a specified goal of the study, the subject group included a wide range of ethnicities and included representation of all Fitzpatrick skin types. This was done to address the reality that many skin therapies will either require careful modulation of parameters or are generally avoided altogether in darker skin types (IV through VI), delineating the utility of an at‐home topical skin therapy that does not require consideration of Fitzpatrick skin type when prescribed. The data was not broken down by skin type due to the sample size; such a breakdown may be revealing if a similar study were performed using a larger sample size with specific attention to this during the recruitment of the subject population. The duration of this study was 12 weeks, while the standard for hyperpigmentation studies is 16 weeks. Further study could determine if the results would be greater if the study were not limited to 12 weeks.

The product is designed to be used alone but works well adjunctively to optimize other common acne scar treatments. The effectiveness of treatments for pitted scars often requires procedural interventions such as micro‐needling with or without radio frequency, lasers, dermabrasion, subcision, TCA cross, or injected fillers, as these help stimulate collagen production or physically fill the indentations. Indented (atrophic) scars typically become more prominent with age as the skin loses its elasticity and volume.

ASG may also provide a ready alternative for patients who, because of skin type or any other reason, cannot or would not choose more aggressive therapies, such as those noted above.

## 5 Conclusions

Acne Scar Gel with Retex‐5 Technology is a safe and effective therapeutic option for improving the appearance of acne scars and related hyperpigmentation. Given the challenge of treating indented scars, a 70% user satisfaction rate with a topical treatment like the ASG would be considered a remarkable outcome. This suggests that while these products may not completely eliminate deep scars, they might provide visible improvements, particularly in skin texture and color. This could enhance the overall appearance. This result likely reflects a combination of perceived improvements in the scars and possibly improvements in the surrounding skin, such as smoother texture, better color evenness, and a more radiant complexion, which can give the skin a healthier look overall.

These findings further suggest that the product effectively improves skin appearance by reducing the appearance of indented acne scars and hyperpigmentation discoloration, while also being well‐tolerated by users. Further study could determine if results would be greater if the study were not limited to 12 weeks. The product may be an ideal first‐line therapy alternative for patients with mild to moderate acne scarring regardless of skin type.

## Disclosure

This study was sponsored by Newmedical Technology Inc., 4065 Commercial Ave, Northbrook, IL, USA. Dr. Katz is a consultant to Newmedical.

## Ethics Statement

The study was conducted under good clinical practice guidelines.

## Consent

Informed consent and photo consent were obtained from all subjects as per the Declaration of Helsinki.

## Conflicts of Interest

The study was sponsored by Newmedical Inc.

## Data Availability

The data that support the findings of this study are available from the corresponding author upon reasonable request.

## References

[1] C. I. Jacob , J. S. Dover , and M. S. Kaminer , “Acne Scarring: A Classification System and Review of Treatment Options,” Journal of the American Academy of Dermatology 45 (2001): 109–117. 11423843 10.1067/mjd.2001.113451

[2] T. Vos , A. D. Flaxman , M. Naghavi , et al., “Years Lived With Disability (YLDs) for 1160 Sequelae of 289 Diseases and Injuries 1990–2010: A Systematic Analysis for the Global Burden of Disease Study,” Lancet 380, no. 9859 (2012): 2163–2196. 23245607 10.1016/S0140-6736(12)61729-2 PMC6350784

[3] P. Duru and Ö. Örsal , “The Effect of Acne on Quality of Life, Social Appearance Anxiety, and Use of Conventional, Complementary, and Alternative Treatments,” Complementary Therapies in Medicine 56 (2021): 102614, 10.1016/j.ctim.2020.102614. 33197675

[4] D. Connolly , H. L. Vu , K. Mariwalla , and N. Saedi , “Acne Scarring‐Pathogenesis, Evaluation, and Treatment Options,” Journal of Clinical and Aesthetic Dermatology 10, no. 9 (2017): 12–23. PMC5749614 29344322

[5] T. A. Mustoe , R. D. Cooter , M. H. Gold , et al., “International Clinical Recommendations on Scar Management,” Plastic and Reconstructive Surgery 110, no. 2 (2002): 560–571. 12142678 10.1097/00006534-200208000-00031

